# 3D Cultures for Modelling the Microenvironment: Current Research Trends and Applications

**DOI:** 10.3390/ijms241311109

**Published:** 2023-07-05

**Authors:** Roberto Gaetani, Isotta Chimenti

**Affiliations:** 1 Department of Molecular Medicine, Faculty of Pharmacy and Medicine, Sapienza University of Rome, 00185 Rome, Italy; 2 Department of Medical and Surgical Sciences and Biotechnology, Faculty of Pharmacy and Medicine, Sapienza University of Rome, 04100 Latina, Italy; 3 Mediterranea Cardiocentro, 80122 Naples, Italy

The importance of 3D culture systems for drug screening or physio-pathological models has exponentially increased in recent years. In our body, cell behaviour is influenced by several factors, ranging from cell–cell contact, cell–extracellular matrix (ECM) interaction, mechanical forces, and growth factor availability among others [1,2,3]. Classical 2D approaches fail to recapitulate the complex in vivo environment, and many potential treatments fail to show a therapeutic effect when tested in vivo, both in small and large animal models. Indeed, to date only a small portion of newly developed drugs are ultimately tested in clinical trials, and only 12% are approved by the FDA for release into the market [4]. At the same time, costs in R&D have increased by about ten times compared to what the industry spent per year in the 1980s, after adjusting for the effects of inflation [4], with large companies investing mainly in marketable drugs, thus limiting the development of therapeutic treatments for less-common diseases. Thus, the development of more complex 3D tissues that better recapitulate the in vivo environment is now emerging as a promising alternative tool for high-throughput drug screening and disease modelling.

Several 3D models are now commonly used, ranging from 3D-engineered tissues to organoids, and even organ-on-chip, as a potential therapeutic product, a model for drug screening or as a method to recapitulate the physiological environment for studying the in vitro human disease mechanism [2,5,6]. Such models could limit the use of animal models, and allow the large-scale testing of drugs at reduced costs, with only the most promising molecules moving forward to clinical trials. The development of these models has been possible thanks to our better understanding of the stimuli important in tissue development and homeostasis. The discovery of induced pluripotent stem cells (iPSCs) has allowed us to recreate human-derived cell types carrying patient mutations, as well as to obtain a large number of differentiated human cells for in vitro studies or cell-based treatments. Similarly, the development of new functionalized biomaterials has allowed us to recreate (at least to some extent) the complex architecture of the ECM, and its important role in molecular signalling and tissue mechanics [7,8,9]. Moreover, tissue printing and new bioreactor designs have allowed us to integrate the spatiotemporal and mechanical cues observed in vivo into small 3D in vitro constructs, thus building a more physiological environment for the cells and the subsequent development of 3D constructs that better mimic the human tissues [2,10].

In this Special Issue, we have gathered scholars in the field of 3D cultures for the creation of in vitro microenvironments for the study of tissue homeostasis and pathology, as we believe that this emerging field is of paramount importance in facilitating our understanding of complex diseases and their pathogenesis, as well as in the development of new therapeutic products. Contributions from multiples perspectives have been collected, from culture optimization to precision medicine, that we will briefly summarize and discuss.

3D culture growth is limited in static cell culture conditions, due to the limited diffusion of nutrients, waste, and oxygen. Bioengineering approaches can support 3D culture efficiency solving these issues. Imashiro et al. [11] introduced a novel system employing diffusion and convection to enhance molecule transportation in a 3D cell culture setting, made of multi-layered cell sheets of normal human dermal fibroblasts. They compared standard dish cultures with those on porous collagen scaffolds with perfusable channels, with or without culture-media perfusion. Their results showed the improved viability of the layered tissue in this perfusion system, with increased glucose consumption, lactate production, and oxygen transport to and by the cells. This setup may improve the viability of thicker artificial 3D tissues, without imposing mechanical stress or damage in the 3D culture.

The control of stem-cell fate represents a compelling aim for cell biology. It is well known that oxygen levels play a key role in cell differentiation in the embryo. Human iPSCs share this susceptibility to oxygen tension, but its early effects on in vitro differentiation is not fully understood. Khadim et al. [12] have exploited 3D human iPSC aggregates cultured on oxygen-permeable microwells under different oxygen concentrations ranging from 2.5 to 20%. They have found a positive correlation between increased oxygen levels and larger aggregate size; conversely, a negative correlation with oxygen was detectable for higher glycolytic pathway activation, and expression of differentiation markers of the three germ layers. Thus, they confirmed that oxygen concentration can function as a regulator of early iPSC differentiation in 3D as a possible cell-fate control strategy.

Relevant in vitro models represent important platforms for the study of many chronic diseases, including degenerative and inflammatory diseases. Primary open-angle glaucoma is a neurodegenerative disorder leading to blindness, with a complex aetiology and largely unknown pathogenesis, involving among others the trabecular meshwork. Preclinical animal models have often failed to mirror clinical studies, evidencing the need for relevant models for drug screening. Vernazza et al. [13] developed an innovative in vitro 3D model of human trabecular cells on a milli-fluidic platform, and they have investigated the effects of pressure and oxidative stress in this advanced glaucoma model. Their results show that oxidative stress has a more prominent role than pressure elevation on trabecular cell damage, and that these stressed cells release signals that enhance apoptosis in neuron-like cells, suggesting a novel pathogenetic mechanism in glaucoma pathogenesis.

Chronic inflammatory diseases are often hard to shape in models. Rheumatoid arthritis involves the destructive processes of cartilage and the subchondral bone, and its pathogenesis is still under study. Damerau et al. [14] have developed an in vitro 3D model of human osteochondral tissue to be able to mimic the cytokine signalling involved in cell and matrix alterations during cartilage and bone degradation. This could provide novel pathogenetic insights on rheumatoid arthritis, as well as serving as an invitro drug screening tool. Mesenchymal stromal cells were studied with bone and cartilage components in a 3D microenvironment, allowing the characterization of different markers. Stimulation with pro-inflammatory cytokines (e.g., TNFα, IL6) induced expression of lactate dehydrogenase A, interleukin-8, and matrix metalloproteases, which was counteracted by treatment with target-specific drugs, preventing inflammation and matrix degradation. Overall, this model may help the preclinical evaluation of new therapeutic targets and novel active molecules in a relevant translational setup.

An important pathogenetic process tightly connected to chronic inflammation is represented by fibrosis. Cardiac fibrosis represents a key pathogenetic step in multiple cardiovascular diseases, and its specific treatment still represents an important unmet clinical need. Dedicated and effective in vitro models are important tools for advancing knowledge on physiopathology and therapy. Picchio et al. [15] reviewed the available literature on 3D multicellular models for the study of cardiac fibrosis using at least two different cell types among cardiac stromal cells, endothelial cells, cardiomyocytes, and/or immune cells, to resemble the complexity of intercellular communication in 3D settings. The review presents examples of spontaneous microtissues, bioprinted constructs, engineered tissues, and organ-on-chip platforms, discussing their advantages and limitations, as well as the discoveries they have provided in terms of physiopathology and screening of novel potential therapeutic molecules.

The use of 3D in vitro models for the study of the tumour microenvironment has risen rapidly in the past years. As previously discussed, the large majority of preclinically selected anti-cancer drugs fail in subsequent clinical trials, mostly due to the oversimplification and limited clinical relevance of in vitro models. Trivedi et al. [16] have reviewed the transformation process of epithelial cancers as they represent 90% of all cases, with the aim of discussing the experimental models available that can simulate their microenvironment, and comparing them to in vivo models. They provided an overview of novel technologies and protocols for tumour spheroids, organoids, and 3D-printed models using both natural and synthetic biomaterials. They also discussed high-content imaging technologies that can be used to investigate the architecture of such 3D models, with a focus on technological advancements and challenges. Finally, they reviewed the potential exploitation of nanotechnological tools for epithelial tumour modelling, and also for the development of innovative nanotherapeutics.

As another example of how 3D cultures can be more representative of the responses to drugs observed in vivo, Filipiak-Duliban et al. [17] investigated the susceptibility of melanoma and renal-cancer-cell spheroids to anti-cancer drugs, including everolimus, doxorubicin, and cisplatin. They describe a tumour-dependent sensitivity, associated with the differential upregulation of the chemo-resistant marker MDR1, and with the differential expression of several metabolism genes. This study highlights the potential of 3D models to uncover non-canonical mechanisms of drug resistance in the tumour microenvironment.

Concerning again the complexity of tumour microenvironments, Wieleba et al. [18] have reviewed the literature on 3D in vitro models of lung cancer, which still represents the most common cancer type. The authors focused on the key factors of lung-cancer biology, its microenvironment, and the current clinical challenges in therapeutic management. The review also summarized the most relevant strategies for in vitro models to assess the tumour microenvironment crosstalk, discussing the advantages and limitations of different settings. Research advancements in this field will facilitate the pre-clinical validation of new therapies to obtain more relevant and personalized results for faster clinical translation.

A very novel field of research concerns the altered ECM and mechanical features of the tumour environment, which appears to oppose immune-cell infiltration as a mechanism of immune-escaping. The abundance of cytotoxic CD8+ cells in the main tumour mass is a predictor of the efficacy of chemotherapy. Chirivì et al. [19] have characterized the phenotypic profiles of CD4+ and CD8+ cells in resting and activated states in a 3D culture system recapitulating the mechanical properties of the tumour, by creating different PEG-fibrinogen-based hydrogels with variable stiffnesses. A high matrix density and stiffness reduced T-cell viability, favouring CD4+ T cells over CD8+ T cells, supporting the notion that the tumour microenvironment might be able to mechanically elude the intervention of cytotoxic lymphocytes. These results shed light on a novel immunosuppressive mechanism within the tumour microenvironment, possibly explaining the correlation between increased mechanical properties in tumours with a worse prognosis in cancer patients.

In conclusion, 3D in vitro models open many possibilities for non-animal research tools, and their translational relevance has been afforded increasing attention. Many challenges still exist, but scientists in this multifaceted and interdisciplinary research field look forward to future exciting developments.

## References

[1] Duval K., Grover H., Han L.H., Mou Y., Pegoraro A.F., Fredberg J., Chen Z. (2017). Modeling Physiological Events in 2D vs. 3D Cell Culture. Physiology.

[2] Murphy S.V., Atala A. (2014). 3D bioprinting of tissues and organs. Nat. Biotechnol..

[3] Pesce M., Messina E., Chimenti I., Beltrami A.P. (2017). Cardiac Mechanoperception: A Life-Long Story from Early Beats to Aging and Failure. Stem Cells Dev..

[4] https://www.cbo.gov/publication/57126.

[5] Ingber D.E. (2022). Human organs-on-chips for disease modelling, drug development and personalized medicine. Nat. Rev. Genet..

[6] Kim J., Koo B.K., Knoblich J.A. (2020). Human organoids: Model systems for human biology and medicine. Nat. Rev. Mol. Cell. Biol..

[7] Trubuil E., D’Angelo A., Solon J. (2021). Tissue mechanics in morphogenesis: Active control of tissue material properties to shape living organisms. Cells Dev..

[8] Pagliarosi O., Picchio V., Chimenti I., Messina E., Gaetani R. (2020). Building an Artificial Cardiac Microenvironment: A Focus on the Extracellular Matrix. Front. Cell. Dev. Biol..

[9] Gaetani R., Zizzi E.A., Deriu M.A., Morbiducci U., Pesce M., Messina E. (2020). When Stiffness Matters: Mechanosensing in Heart Development and Disease. Front. Cell Dev. Biol..

[10] Bhatia S.N., Ingber D.E. (2014). Microfluidic organs-on-chips. Nat. Biotechnol..

[11] Imashiro C., Yamasaki K., Tanaka R.I., Tobe Y., Sakaguchi K., Shimizu T. (2021). Perfusable System Using Porous Collagen Gel Scaffold Actively Provides Fresh Culture Media to a Cultured 3D Tissue. Int. J. Mol. Sci..

[12] Khadim R.R., Vadivelu R.K., Utami T., Torizal F.G., Nishikawa M., Sakai Y. (2022). Integrating Oxygen and 3D Cell Culture System: A Simple Tool to Elucidate the Cell Fate Decision of hiPSCs. Int. J. Mol. Sci..

[13] Vernazza S., Tirendi S., Passalacqua M., Piacente F., Scarfi S., Oddone F., Bassi A.M. (2021). An Innovative In Vitro Open-Angle Glaucoma Model (IVOM) Shows Changes Induced by Increased Ocular Pressure and Oxidative Stress. Int. J. Mol. Sci..

[14] Damerau A., Pfeiffenberger M., Weber M.C., Burmester G.R., Buttgereit F., Gaber T., Lang A. (2020). A Human Osteochondral Tissue Model Mimicking Cytokine-Induced Key Features of Arthritis In Vitro. Int. J. Mol. Sci..

[15] Picchio V., Floris E., Derevyanchuk Y., Cozzolino C., Messina E., Pagano F., Chimenti I., Gaetani R. (2022). Multicellular 3D Models for the Study of Cardiac Fibrosis. Int. J. Mol. Sci..

[16] Trivedi P., Liu R., Bi H., Xu C., Rosenholm J.M., Akerfelt M. (2021). 3D Modeling of Epithelial Tumors-The Synergy between Materials Engineering, 3D Bioprinting, High-Content Imaging, and Nanotechnology. Int. J. Mol. Sci..

[17] Filipiak-Duliban A., Brodaczewska K., Kajdasz A., Kieda C. (2022). Spheroid Culture Differentially Affects Cancer Cell Sensitivity to Drugs in Melanoma and RCC Models. Int. J. Mol. Sci..

[18] Wieleba I., Wojas-Krawczyk K., Krawczyk P., Milanowski J. (2022). Clinical Application Perspectives of Lung Cancers 3D Tumor Microenvironment Models for In Vitro Cultures. Int. J. Mol. Sci..

[19] Chirivi M., Maiullari F., Milan M., Presutti D., Cordiglieri C., Crosti M., Sarnicola M.L., Soluri A., Volpi M., Swieszkowski W. (2021). Tumor Extracellular Matrix Stiffness Promptly Modulates the Phenotype and Gene Expression of Infiltrating T Lymphocytes. Int. J. Mol. Sci..

